# Spore-Trapping Device: An Efficient Tool to Manage Fungal Diseases in Winter Wheat Crops

**DOI:** 10.3390/plants12020391

**Published:** 2023-01-14

**Authors:** Oksana Kremneva, Roman Danilov, Ksenia Gasiyan, Artem Ponomarev

**Affiliations:** Federal State Budgetary Scientific Institution «Federal Research Center of Biological Plant Protection» (FSBSI FRCBPP), Krasnodar 350039, Russia

**Keywords:** winter wheat, wheat diseases, spore-catching device, disease development, spores, weather conditions, cultivar

## Abstract

Leaf airborne diseases cause major shortfalls in agricultural crops. The introduction of technical means can significantly improve early-warning systems for plant diseases as well as provide timely and accurate forecasts. In this paper, we aim to evaluate the possibilities of detecting a phytopathogenic infection using a spore-catching device developed at the Federal Research Center of Biological Plant Protection (FRCBPP) on winter wheat varieties of different levels of susceptibility to major economically important leaf diseases, taking into account climatic conditions. The device captures spores in the surface layer of air among crop plants. We conducted research in the experimental fields of FRCBPP in 2019–2021. The objects of the study were four cultivars of winter wheat. They were selected according to the degree of resistance to various leaf diseases. We studied the progress of wheat diseases according to generally accepted international scales the onset of the first manifestations to their maximum development. We studied the aerogenic infection in wheat crops using the FRCBPP developed portable device for determining the infestation of plants. Sampling was carried out in the same period as the visual assessment. The samples were taken in the crops of each variety at five points. The sampling time was one minute. As a result of research on experimental crops of four varieties of winter wheat, we observed the development of such diseases as powdery mildew (*Erysiphe graminis*), yellow spot (*Pyrenophora tritici-repentis*), septoria leaf spot (*Septoria tritici*), yellow *(Puccinia striiformis)* and brown rust (*Puccinia triticina)*. In a laboratory study of samples under a light microscope, all of the listed pathogens were found except for septoria leaf spot. Two-way analysis of variance confirmed the statistically significant separate and cumulative influence of the cultivar and year factor on winter wheat diseases. A generalized correlation analysis for three growing seasons (2019–2021) showed that an average statistically significant correlation coefficient (0.5–0.6) remains for the total groups for powdery mildew, yellow and brown rust. This indicator for the causative agent of yellow spot was equal to 0.4 with a high level of statistical significance. Thus, we conclude that by using a spore-catching device, it is possible to identify spores of economically significant pathogens in winter wheat crops and predict the further development of pathogens, taking into account the cultivar and annual climate factors.

## 1. Introduction

Leaf diseases, transmitted through airborne fungi, are the cause of significant shortfalls in agricultural crops. For wheat, global losses from leaf rust, yellow rust and yellow spot reach up to 15–40%; from stem and yellow rust—up to 40–90% [1,2,3,4]. In the Russian Federation in 2021, wheat sown area amounted to 28.72 million hectares. Such large areas require regular phytosanitary monitoring. It helps to improve early-warning systems for plant diseases, provide timely and accurate forecasts. Classical visual evaluation requires a lot of labor resources and is not always accurate due to large production areas. The observed climate change contributes to the growth, virulence, reproduction and expansion of the range of the most prominent wheat diseases [3,5].

The introduction of technical means in agriculture can significantly improve early-warning systems for plant diseases as well as provide timely and reliable forecasts. In this regard, air samplers that are used to detect and monitor airborne infections can be seen as a key implementation [6].

In world practice, stationary spore collectors by Burkard, Lanzoni and Rotorod are used for monitoring for various purposes. An aeropalynologic survey of the atmosphere of La Plata, Argentina was carried out using a Lanzoni volumetric spore trap: seventy-nine morphological types of spores of different taxa were identified [7]. Burkard volumetric spore traps were used to study seasonal abundance of *Botryosphaeriaceae* spp. spores in California vineyards [8], as well as to detect airborne sporangia of *Pseudoperonospora cubensis* and *P. humuli* in Michigan [9]. Rotorod spore trap was used to quantify the potential spread of *Phytophthora infestans* spores over US potato fields [10]. There are some studies that prove the highest efficiency of portable spore catchers compared to the stationary ones [11].

France is developing various methods of air sampling, as well as improving spore traps in the context of plant biosecurity. The Coriolis δ air sampler manufactured by Bertin Technologies (France) is a continuous air sampler. It is based on patented Coriolis technology delivering a liquid sample. This, in turn, allows express tests by several methods, including PCR and serological analyses [12]. In Australia, scientists have identified several problems regarding the use of air samplers and have proposed a number of improvements [13]. The National Institute for Occupational Safety and Health (NIOSH, USA) develops a personal sampler for collecting fungal spores, the principle of operation of which is similar to the French “Coriolis δ” [14].

In Russia, the Federal Research Center of Biological Plant Protection (FRCBPP) develops and studies phytosanitary monitoring devices. The devices “Determinant of plant infestation OZR-1mp” [15,16] and “Air sampler PSL” have been developed. The devices are designed to detect spores of phytopathogens of grain crops. The device “Determinant of plant infestation OZR-1mp” detects pathogen spores in the surface air layer among crop plants. “Air sampler PSL” can be used in combination with unmanned aerial vehicles such as multicopters and thus can provide phytosanitary monitoring over long distances and in hard-to-reach places [17]. Kazan State Agrarian University has created its own version of a spore catcher “Spore catcher for collecting the infectious beginning of phytopathogenic fungi from the surface of plants”—in it, spores through special trapping holes remain on the surface of the plant. After 1–7 days, the device, together with the plant site, is transferred to the laboratory and further studies are carried out to count and identify spores. The spore catcher helps to determine the infectious load or the number of spores of phytopathogenic fungi per unit of plant surface [18].

The weather factor in the development of the diseases of any crops is very significant, since the spread (release of spores) and the development of airborne fungi depend on certain weather conditions [19]. However, the infection and the course of the disease depend on the susceptibility of the plant on which the spores were able to settle and gain a foothold. Therefore, the cultivar is also an important factor in the development of protective measures.

In this study, we aim to evaluate the possibilities of detecting a phytopathogenic infection using a spore-catching device developed at FRCBPP on winter wheat varieties of different levels of susceptibility to major economically important leaf diseases, taking into account climatic conditions.

## 2. Results

A visual assessment during the research period from 2019 to 2021 on experimental winter wheat crops resulted in the confirmation of the development of the following diseases: powdery mildew, yellow spot, septoria leaf spot, yellow and brown rust (Table 1). We used a spore-catching device and detected spores of all the listed pathogens except for septoria leaf spot. The reason lies in the fact that the spread of this pathogen occurs mainly with dripping moisture (rain, dew), and for technical reasons, the device is operational only in dry weather.

At the first key moment of winter wheat vegetation (phase GS 40–47 “flag leaf”), two-way analysis of variance confirmed the statistically significant separate and cumulative influence of cultivar and year factors on the development of powdery mildew, septoria leaf spot and yellow rust pathogens (Table 2). The influence of these factors was not confirmed for yellow spot and leaf rust.

In 2019, the minimum number of powdery mildew spores was recorded, which varied from 5 to 20 in different cultivars. External signs were not detected at all, since the weather conditions that year turned out to be unfavorable for the development of the pathogen.

In 2020 and 2021, more favorable for the pathogen, the average indicators of the disease development varied from 2 to 3% with the number of spores of 20–40 pcs and up to 3–5% with the number of spores of 40–60 pcs, respectively. At the same time, cvs Kuren and Aksinya were characterized by the maximum indicators, and cvs Bonus and Krasnodarskaya 99 were characterized by the minimum ones. This generally corresponds to the resistance of cultivars to the disease.

Spores of other pathogens in the GS 40–47 phase during three years of research were noted in the amount of 1–2 pcs. Sole manifestations of yellow rust were observed in 2019 on all varieties. There was also a sole manifestation of yellow spot in 2020 and 2021.

Correlation analysis showed that due to the minimal manifestation of diseases and a single number of spores for most variants of the experiment, the relationship between these indicators was insignificant or completely absent (Table 3). A positive and statistically significant relationship was determined for yellow spot on cv Aksinya, which is the most susceptible to this pathogen, as well as for yellow rust on the total group of varieties, among which cv Bonus stood out with the highest correlation of 0.9.

During the second vegetation period (phase GS 61 “early flowering”), we noted the beginning of the rapid development of all diseases (Table 1).

Two-way analysis of variance confirmed the statistically significant separate and cumulative influence of cultivar and year conditions for powdery mildew, leaf rust, septoria leaf spot and yellow rust pathogens (Table 2). The influence of the year factor was confirmed only for the yellow spot.

In 2019, during this phase, powdery mildew occurred once, and the average number of spores increased to 90 pcs (Table 1). Pathogen development in 2020 reached 7–8% with a spore count of 110–160 pcs for cvs Kuren, Aksinya and Krasnodarskaya 99. A minimum disease development of 5% with a spore count of 80 pcs was noted on cv Bonus. A different situation was observed in 2021, when the maximum pathogen development of 5–7% with the number of spores of 20–70 pcs was noted on the cvs Kuren and Bonus, and the minimum 1–2% on the cvs Aksinya and Krasnodarskaya 99; at the same time, the average indicators of the number of spores were higher than on the cvs Kuren and Bonus from 70 to 80 pcs. This is due to the weather influence in a specific time period, or the peculiarity of the immune response of cultivars to the disease.

Correlation analysis revealed an average level of statistically significant dependence of the number of trapped spores and the degree of development of powdery mildew equal to 0.5 for the total group of cultivars (Table 3). In the total group of cultivars, cv Krasnodarskaya 99 stood out with a fairly high correlation value of 0.7.

In 2019, during this phase, powdery mildew manifested only once, and the average number of spores increased to 90 pcs. The pathogen development in 2020 reached 7–8% with the number of spores 110–160 pcs for cvs Kuren, Aksinya and Krasnodarskaya 99. The minimum development of the disease was 5% with the number of spores 80 pcs was noted on cv Bonus. A different situation was observed in 2021, when the maximum development of the pathogen of 5–7% with the number of spores of 20–70 pcs was registered on cvs Kuren and Bonus, and the minimum 1–2% on cvs Aksinya and Krasnodarskaya 99; the average numbers of spores was higher than on cvs Kuren and Bonus from 70 to 80 pcs. Such a phenomenon could be a result of weather factors in a specific time period, or a specific immune response of the cultivars to the disease. Correlation analysis revealed an average level of statistically significant dependence of the number of trapped spores and the degree of development of powdery mildew equal to 0.5 for the total group of cultivars (Table 3). In the total group of cultivars, cv Krasnodarskaya 99 stood out with a fairly high correlation value of 0.7.

A single manifestation of leaf rust was registered solely in 2019 on the pathogen susceptible cv Krasnodarskaya 99 (Table 1). The number of recorded pathogen spores was 20 pcs. Rather high and statistically significant correlation, equal to 0.8, of the development of leaf rust with the number of spores was found for the above-mentioned cultivar. We observed no external signs of disease manifestation during the three years of research on all other cultivars, the number of detected spores varied from 2 to 6 pcs.

Yellow rust manifested in 2019 and 2020 mainly on cvs Kuren and Bonus, which are the most susceptible to this pathogen. In 2019, the disease development was 1% with the number of spores from 2 to 8 pcs (Table 1), but in 2021, it reached 2–3% with the number of spores 1–2 pcs. In 2020, it was not possible to detect any external signs of the development of the disease or sporulation. We conducted a correlation analysis of the relationship between the number of spores and the development of the pathogen for cvs Kuren and Bonus. Thus, a correlation coefficient was obtained with a high statistically significant indicator of 0.8, and for the total group of cultivars—0.6 (Table 3).

The maximum development of septoria leaf spot with an average of 17.4% was observed on cv Kuren in 2019 (Table 1). On the rest of the cultivars this year, the disease progression rate varied between 8 and 10%. In 2020, the disease did not manifest; in 2021 it reappeared, but with less intensity. The highest manifestation with a mean value of 3.5% was also observed on cv Kuren. The rest of the cultivars were between 0.5 and 1%.

Yellow spot manifested in the GS 61 “early flowering” phase only in 2019 with development up to 1% (Table 1). At the same time, the number of spores, as in the previous moment of time, was single, only 1–2 pcs. In 2020 and 2021, the disease was not detected. However, the correlation analysis revealed a high correlation between the number of spores and the development of the disease, equal to 0.9 per cvs Kuren and Krasnodarskaya 99. Thus, a correlation coefficient was obtained with a high statistically significant indicator of 0.6 for the total group of cultivars (Table 3).

At the third growing season (end of May, beginning of June, phase GS 71–82 “milk-wax ripeness”), the development of pathogens increased significantly (Table 1). A two-way analysis of variance confirmed statistically significant separate and cumulative influence of the cultivar and weather factors for pathogens of powdery mildew, septoria leaf spot, yellow and brown rusts (Table 2). However, the influence of these factors was not confirmed for yellow spot. The development of powdery mildew in 2019 was 1–3%, and the number of spores varied from 20 to 60 pcs. In 2020 and 2021, the development of the disease reached 15–20%. The number of spores has also increased to 200–400 pcs. A high correlation level of the disease development and the number of spores was revealed: 0.8–0.9 for individual cultivars, and 0.7 for the total group (Table 3).

In 2019, during this period, the maximum intensity of brown rust was observed with an average of 10.3% on the pathogen susceptible cv Krasnodar 99 with the largest number of spores 21 pcs (Table 1). A one-time manifestation of the disease was noted with the number of spores from 1 to 5 pcs on other cultivars. The year 2020, which was unfavorable for the pathogen, was characterized by a single manifestation of the disease on cv Krasnodarskaya 99 with the number of spores 1–2 pcs. In 2021, the development of the disease on cv Krasnodarskaya 99 was 4.5%, and the number of spores was 58 pcs. Therefore, the highest level of correlation between the data on the development of leaf rust and the number of recorded pathogen spores (0.9) was found on cv Krasnodarskaya 99. This indicator was 0.7 for cvs Kuren and Aksinya, and for the total group—0.8 (Table 3).

Yellow rust development was observed in 2019 and 2021. In 2019, the disease reached 3.5% on cvs Kuren and Bonus. At the same time, the average number of pathogen spores remained at the previous level and varied from 1 to 9 pcs (Table 1). The maximum intensity of yellow rust occurred in 2021, when the average development rate of the disease was 8.8% on cv Bonus, and the number of recorded spores was 17 pcs.

The level of correlation between disease progression data and spore count for yellow rust was high: 0.8–0.9 both for the total group and for individual cultivars (Table 3).

The manifestation of septoria leaf spot slightly decreased compared to the previous growing season. The maximum development of the disease of 12.3% was observed in 2021 on cv Bonus, and the minimum of 2.4% in 2020 and 2021 on cv Aksinya (Table 1). In 2020, the disease development was not observed.

Yellow spot was also registered in 2019 and 2021. In 2019, the disease intensity varied from 3.5 to 4.3%, reaching a maximum at cvs Aksinya and Krasnodarskaya 99 (Table 1). The number of pathogen spores varied from 1 to 3 pcs. It is noteworthy that in 2021 there was a single manifestation of the pathogen with a development of up to 1% and spores were recorded in an amount from 1 to 8 pcs. The correlation analysis of the disease development and the number of spores showed a high correlation level of 0.9 only on cv Krasnodarskaya 99. Meanwhile, a low but statistically significant indicator (0.4) was obtained for the total group (Table 3).

A generalized correlation analysis for three growing seasons (2019–2021) showed that an average statistically significant correlation coefficient (0.5–0.6) remains for the total groups for powdery mildew, yellow and brown rust (Table 3). Cv Krasnodarskaya 99 had a high correlation coefficient (0.8) for powdery mildew, and cvs Kuren and Bonus had an average value of 0.5–0.6. Cvs Kuren and Bonus had a high correlation coefficient (0.8). Correlation coefficient for leaf rust equal to 0.7 was noted for cv Krasnodarskaya 99. For yellow spot for the total group of cultivars, correlation coefficient was rather weak—0.4, still, having a high level of statistical significance. For individual cvs Kuren and Krasnodarskaya 99, only an average correlation of 0.5–0.6 was noted.

## 3. Discussion

As a result of research on experimental crops of four varieties of winter wheat, we observed the development of such diseases as powdery mildew, yellow spot, septoria leaf spot, yellow and brown rust. Using a device for rapid spore detection, we were able to trace the sporulation of all the listed pathogens, except for septoria leaf spot.

Two-way analysis of variance confirmed the statistically significant separate and cumulative influence of the cultivar and year factor on winter wheat diseases.

According to the literature data, the most favorable conditions for pathogens of powdery mildew (*E. graminis*), yellow rust (*P. striiformis*) and septoria leaf spot (*S. tritici*) are temperatures of 9–15 °C, 12–20 °C and 18 °C, respectively, and high humidity that occurs during precipitation, or with a sharp alternation of air temperatures, provoking the precipitation of an abundant amount of dew. Therefore, these diseases are characterized by the early onset of spore release and the manifestation of symptoms on plants (April–May).

In our study, this fact for powdery mildew was confirmed in 2019 and 2020, when the peak of spore release of the causative agent, as well as the largest percentage of the disease development occurred in early-mid May. However, in a colder and wetter 2021, powdery mildew peaks have shifted to the end of May. The influence of weather factors on wheat yellow rust has also been confirmed. In 2019, both the peaks of spore release and the disease development took place in mid-late May. In 2020, this disease and pathogen spores were not detected. This is due to low rainfall in March and April, insufficient for the development and spread of the disease.

In 2021, the peak of spore release as well as disease development were noted in late May–early June, which is also associated with lower temperatures this year and higher humidity. The influence of weather factors on septoria leaf spot was also confirmed. In 2019, the first peak in the development of the disease was noted in mid-May. Then it sharply increased in late May–early June. This, apparently, is due to the fact that favorable conditions for the phytopathogen *S. tritici* at early stages allowed it to securely attach itself to leaf blades and continue its active development in a later period.

Septoria leaf spot in 2020 was not distributed to cvs Aksinya and Krasnodarskaya 99. On cv “Bonus”, the minimum development (1%) was noted in mid-May, and on cv “Kuren”, which is the most susceptible to this disease, the highest degree of development (10%) was noted in early June. This may be due to a sharp increase in rainfall in May 2020 and lower temperatures, which created optimal conditions and allowed the development of the pathogen. The year 2021 was the most favorable for septoria leaf spot, since the optimal climate for this disease was observed almost throughout the entire growing season. Consequently, the first detected symptoms of the disease in mid-May had already received a high degree of development (5–15%) and subsequently reached 15–26% by early June.

The optimal environmental conditions for the development and spread of the causative agent of leaf rust (*P. triticina*) and yellow spot (*P. tritici-repentis*) are temperatures of 20–25 °C and prolonged moisture. Therefore, the infection spreads later (May–June). It has been established that, in addition to climate factors, the development and spread of leaf rust is significantly influenced by the cultivar factor.

The spore release of the causative agent of this disease was noted throughout May in 2019. However, we did not detect this disease on cv “Kuren” resistant to it. The disease intensity did not exceed 1% for Bonus and Aksinya cvs, which are moderately resistant and moderately susceptible, respectively. At the same time, the disease manifested in late May. Cv “Krasnodarskaya 99” was the only one susceptible to leaf rust, so it can be clearly traced the influence of weather factors on the development of this disease. The highest degree of development (33%) was noted in late May–early June 2019, which corresponds to the literature data on the optimal conditions for the pathogen.

The spore release of the leaf rust pathogen was also observed throughout May in 2020. However, in quantitative terms, infectious particles were found significantly less than in the previous year—no more than 20 pcs for the entire season (in 2019, for comparison, it was found from 20 up to 120 pcs). This indicates unfavorable conditions for active sporulation of *P. triticina*. The spore release peaked in mid-late May.

The highest degree of infestation was registered on cv “Krasnodarskaya 99” (no manifestation on other cultivars) in late May–early June and did not exceed 1%. Low temperatures prevented the manifestation of the disease in mid-May 2021. The beginning of sporulation was recorded only with an increase in temperatures in early June, at the same time, brown rust pustules were found on leaf blades of cv “Krasnodarskaya 99”. Moreover, the degree of manifestation was quite high—from 10% to 14%.

The influence of weather conditions and the cultivar factor on the development of yellow spot is not so clear. In 2019, the peak of spore release was set in mid-May, and the highest damage percentage—at the end of May. In 2020, it was not possible to establish the peak of spore release: for the entire season, only four pcs of spores of the causative agent of this disease were found over all crops, while the degree of infestation on all cultivars did not exceed 1%. Thus, 2020 was the most unfavorable for yellow spot. In general, taking into account the weather factor, in 2019–2020, the disease development falls within the definition of the norm.

Atypical development and spread of the disease were observed in 2021. The first symptoms on all cultivars were noted in early May (1–3%). Then, for a month, no further development was detected, and in early June, the degree of damage increased sharply, ranging from 3% to 5%. The peak of spore release was recorded at the end of May–beginning of June.

This development may be due to several reasons. Firstly, *P. tritici-repentis* can thrive in a wide temperature range from 5 °C to 35 °C. Secondly, in addition to weather and cultivar factors, aspects such as tillage and previous crops may have influenced the development and spread of the fungus. Since the presence of stubble, which is the place of wintering and further spread of the infectious onset, determines the degree of development of this disease.

Thus, the conducted studies allow us to conclude that it is possible to detect diseases at the initial stage on winter wheat crops using a spore-catching device. This permits to predict the further development of pathogens, taking into account the cultivar and climate factors.

The analysis of the data of the second growing season made it possible to identify the average level (0.5–0.6) of the correlation between the development of pathogens and the number of spores at a high level of statistical significance. However, the most important is the fact that the data obtained confirm the possibility of predicting the development of pathogens 10 or more days before the external signs of their manifestation on winter wheat crops.

In general, in the third growing season for most pathogens, with the exception of powdery mildew, there was no significant increase in the dynamics of sporulation. Nevertheless, we obtained high degrees of data correlation due to the increase in the intensity of their development. This indicates that the degree of development of diseases and the quantitative composition of spores correspond to a high level of statistical significance.

Studying the dynamics of spore release of phytopathogenic fungi using spore trapping devices to monitor plant diseases is a fairly popular direction [20,21,22]. At the same time, the development of sampling technologies is aimed at improving the capture efficiency at relatively high speeds of collecting air volume, simplifying sample processing (since the main method is the subsequent analysis of the obtained particles using a microscope), installing air samplers on mobile platforms, such as UAVs for automation and achievement of efficiency of sampling [21]. The use of GIS made it possible to more accurately predict the airborne inoculum and track the distribution dynamics [6].

Our study is unique in that it shows the correlation between the number of phytopathogen spores detected and the actual development of diseases, while taking into account the characteristics of cultivar and climatic to build a development forecast system at the initial stage of monitoring. We assume that it is crucial for subsequent practical applications.

In the literature review, we mentioned spore-catching devices used in aerobiological research around the world [6,7,8,9,10,11,12,13,14,15]. There are examples of using these devices to monitor the flight of pathogen spores on a number of agricultural crops (coffee, soybeans, and sugarcane) [8,9,10,11,17]. The device that we tested in our studies is designed to analyze the concentration of spores in the surface layer of air among plants in cereal crops.

## 4. Materials and Methods

We conducted research in the experimental fields of the Federal Research Center of Biological Plant Protection, Krasnodar (FRCBPP) (45°2.413′0″ N, 38°58.5598′0″ E, 29 m a.s.l.) from 2019 to 2021. The objects of the study were four cultivars of winter wheat. They were selected according to the degree of resistance to various diseases [23,24]: “Kuren” (resistant to brown and yellow rust, moderately resistant to powdery mildew, moderately susceptible to septoria and yellow spot), “Bonus” (resistant to leaf rust and powdery mildew, moderately susceptible to septoria leaf spot, susceptible to yellow rust and yellow spot), “Aksinya” (resistant to leaf rust, moderately resistant to yellow rust, moderately susceptible to powdery mildew and septoria leaf spot, susceptible to yellow spot), “Krasnodarskaya 99” (resistant to yellow rust, moderately resistant to powdery mildew and septoria leaf spot, moderately susceptible to yellow spot, susceptible to leaf rust). The plot area of each cultivar was 20 m^2^ in triplicate.

We studied the progress of wheat diseases according to generally accepted international scales: the Peterson scale to estimate the intensity of rust diseases [25]; the Saari-Prescot for yellow spot [26]; special CIMMYT developed scales—powdery mildew and septoria leaf spot [27].

The counts were carried out from the onset of the first manifestations of diseases (beginning of May) to their maximum development (beginning of June). At the same time, three key moments of vegetation were identified, timed to specific stages of ontogeny of winter wheat plants:

- The first key time period of vegetation, considered during the three years of research from 2019 to 2021, is timed to the beginning of May, when wheat plants reach the GS 40–47 phase (Zadoks scale) [28] “flag leaf”. This point in time promotes an early detection of the infectious onset at the initial stages of pathogen development.
- The second growing season sets up at the second decade of May, when the winter wheat plants reach the GS 61 “early flowering” phase. At this time, all leaf-stem diseases are intensively manifested. This time period is an important link for compiling a predictive model for the development of pathogens. As it allows a comparative analysis of the quantitative indicators of their development (degree of development, number of spores) from the moment of primary signs (after the incubation period) to the onset of intensive manifestation, taking into account the influence of cultivar and weather factors of a given year.
- The third period under consideration is the end of May, the beginning of June. This is the phase GS 71–82 “milk-wax ripeness” of winter wheat. The data obtained at this point in time are the final logical component of the research and can become a potential basis for predicting yields.

**Figure 1:**
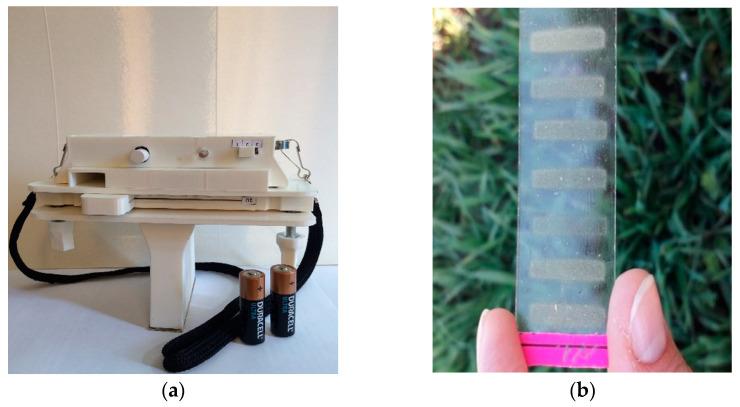
The device used in the study of aerogenic infection in wheat crops: (**a**) portable device for determining the infestation of plants; (**b**) sample prints.

We studied the aerogenic infection in wheat crops using the FRCBPP developed portable device for determining the infestation of plants [29]. The device is an impactor, inside of which there is a glass slide with a retaining composition (vaseline) applied, into which spores of phytopathogenic fungi settle in the form of rectangular imprints with an area of 100 mm^2^ (Figure 1).

**Figure 2:**
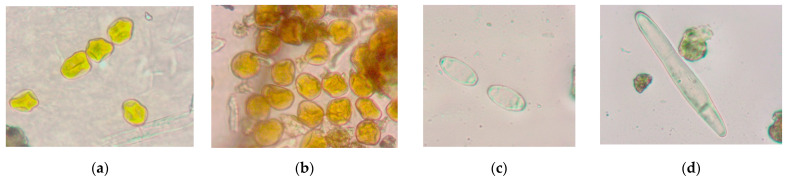
Spores of pathogens found in crops: (**a**) *P. striiformis*; (**b**) *P. triticina*; (**c**) *E. graminis*; (**d**) *P. tritici-repentis*.

Sampling was carried out in the same period as the visual assessment in the winter wheat varieties described above. The sampling time was one minute. When sampling, the device was lowered on a belt into the herbage so that the crevice nozzle was 8–10 cm below the plant tops. After turning on the device, the aspirator located in the device case starts. Further, giving the device oscillatory movements with an amplitude of 15–20 cm, sampling was carried out at five points of each plot with winter wheat. This method of taking air among plants helps to shake off spores from leaves and stems and involve them in the aspiration zone. After the end signal, we turned off the device and moved on to the next point. Subsequent samples were taken after moving the slide from right to left to a new position (Figure 1b). After sampling, slides are analyzed under a microscope to identify and count spores (Figure 2).

To detect, identify and quantify phytopathogenic fungi, the obtained samples were examined under a light microscope at a 10x objective magnification. To count the spores in the sample, a test strip was examined, which fell into the field of view in the center of the sample along the larger side. Then we found out the required viewing area: if the number of spores in the test strip was 0, 1–3, 4–7, >8, then the required viewing area (s) was 100%, 60%, 40%, 20%, respectively [30,31].

Next, the number of strips (*k*) necessary for viewing at the selected microscope magnification was determined using the following formula (1):

(1) $$k=\frac{S}{20\cdot d}$$

where *k* is the number of strips to view, pcs.;

*s* is the minimum required area to be viewed, %;

*d* is the diameter of the field of view, mm;

20 is the length of the scanned strip, mm.

We then counted the spores and determined the phytopathogens on the set number of strips. Next, their total number was determined by the formula (2):

(2) $$N=\frac{5n}{d\cdot k},$$

where *N* is the number of spores on the entire imprint, pcs.;

*n* is the number of spores in k strips, pcs.;

*d* is the diameter of the field of view of the microscope, mm.

**Figure 3:**
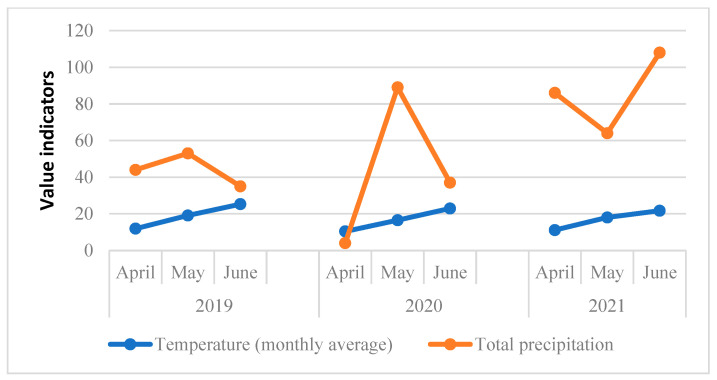
Average monthly air temperatures and total precipitation in 2019–2021 during the study period.

The climate in 2019 compared to 2020 and 2021 was characterized by higher temperatures (Figure 3). Humidity in April 2019 and 2021 was higher than in 2020, which promoted the development of brown and yellow rust. However, the humidity in May 2020 increased sharply due to intense precipitation. Thus, 2020 became more favorable for the development of powdery mildew.

### Statistical Analysis

Statistical data processing was applied in the Statistica 2010 program to analyze and compare the obtained results. We used the method of two-factor analysis of variance to assess the influence of cultivar and climate factors on disease development. The nature of the distribution of the variable values of the obtained data was significantly different from the normal Gaussian distribution. Therefore, the correlation analysis of the relationship between the number of spores caught using a trapping device and the degree of disease development was carried out on the basis of non-parametric statistics methods using the Spearman correlation at a high 95% significance level.

## 5. Conclusions

As a result of the study (2019–2021) on experimental winter wheat crops using a spore-trapping device, we recorded sporulation of pathogens of major leaf diseases, except for septoria leaf spot.

The influence of weather and cultivar factors on the development and spread of fungal leaf diseases has been established and statistically proven.

The obtained results indicate the possibility of their application for compiling a predictive model for the development of pathogens in relation to the use of a spore-catching device or other similar spore-catching equipment.

## Figures and Tables

**Table 1:** Average indicators of disease development and the number of spores on winter wheat crops for three growing seasons (2019–2021).

Disease/ Disease Development, %/ Number of Spores, pcs		Cultivar											
		Kuren			Bonus			Aksinya			Krasnodarskaya 99		
		2019	2020	2021	2019	2020	2021	2019	2020	2021	2019	2020	2021
Phase GS 40–47 “flag leaf” 1.05–2.05													
Powdery mildew	R,% *	0	5.4	3.8	0	3.2	2.4	0	5.6	3.1	0	2.2	1.7
	N,pcs **	19	60	63	5	40	118	19	60	48	13	43	16
Brown rust	R,% *	0	0	0	0	0	0	0	0	0	0	0	0
	N,pcs **	1	1	0	0	1	0	2	1	0	3	0	0
Yellow rust	R,% *	0.2	0	0	0.2	0	0	0.2	0	0	0.2	0	0
	N,pcs **	2	0	1	2	1	0	3	0	0	2	0	0
Septoria	R,% *	0	0	0	0	0	0	0	0	0	0	0	0
	N,pcs **	0	0	0	0	0	0	0	0	0	0	0	0
Pyrenophorosis	R,% *	0	0	0.1	0	0.1	0.3	0	0.1	0.5	0	0	0.7
	N,pcs **	1	0	0	1	0	0	2	0	2	1	0	1
Phase GS 61 “early flowering” 12.05–14.05													
Powdery mildew	R,% *	0.1	7.3	7.9	0.1	5.0	5.0	0.1	7.6	1.6	0.1	7.0	0.3
	N,pcs **	40	135	67	23	80	21	69	159	80	92	108	68
Brown rust	R,% *	0	0	0	0	0	0	0	0	0	0.2	0	0
	N,pcs **	1	2	0	6	2	0	1	4	0	20	2	0
Yellow rust	R,% *	1.0	0	2.2	1.0	0	2.4	1.0	0	0.5	0.6	0	0
	N,pcs **	2	0	1	2	0	2	8	0	0	1	0	0
Septoria	R,% *	17.4	0	3.5	8.5	0	0.8	8.9	0.1	1.0	9.5	0	0.4
	N,pcs **	0	0	0	0	0	0	0	0	0	0	0	0
Pyrenophorosis	R,% *	0.4	0	0	1.0	0	0.1	0.3	0	0	0.3	0	0
	N,pcs **	1	0	0	1	0	0	2	0	0	1	0	0
Phase GS 71–82 “milk-wax ripeness” 24.05–25.05													
Powdery mildew	R,% *	3.1	9.5	15.8	3.0	5.4	7.9	2.8	6.2	3.9	1.3	5.4	0.1
	N,pcs **	20	179	336	21	242	298	32	244	434	60	139	155
Brown rust	R,% *	0.1	0	0.6	0.1	0	0	0.3	0	0	10.3	0.2	4.1
	N,pcs **	1	1	12	5	1	1	3	1	1	21	2	58
Yellow rust	R,% *	3.0	0	2.4	3.4	0	8.8	2.7	0	4.8	0.7	0	0.2
	N,pcs **	9	0	3	1	0	17	9	0	2	8	0	1
Septoria	R,% *	6.1	0	3.8	6.0	0	12.3	2.4	0	2.4	5.3	0	4.9
	N,pcs **	0	0	0	0	0	0	0	0	0	0	0	0
Pyrenophorosis	R,% *	3.6	0	0.1	3.5	0	1.0	4.3	0.1	0.4	4.0	0.1	0.1
	N,pcs **	2	0	1	1	0	2	1	0	2	3	0	8

*—average progression of the disease % **—number of pathogen spores, pcs.

**Table 2:** Assessment of the influence of the factors cultivar and year on the development of winter wheat pathogens for three growing seasons from 2019 to 2021.

Factor	Powdery Mildew			Yellow Spot			Septoria			Yellow Rust			Brown Rust		
	F	F crit.	*p*	F	F crit.	*p*	F	F crit.	*p*	F	F crit.	*p*	F	F crit.	*p*
Phase GS 40–47 “flag leaf” 1.05–2.05															
Cultivar	8.9 *	2.61	0.00008 *	1.2	2.61	0.294	2.8 *	2.61	0.04 *	5.7 *	2.61	0.0007 *	0.6	2.61	0.605
Year	31.8 *	2.64	0.00000 *	1.4	2.64	0.243	27.7 *	2.64	0.00000 *	20.1 *	2.64	0.00000 *	0.7	2.64	0.497
Cultivar * Year	8.5 *	2.61	0.00000 *	1.4	2.61	0.205	1.2	2.61	0.280	4.5 *	2.61	0.0002 *	0.7	2.61	0.649
Phase GS 61 “early flowering” 12.05–14.05															
Cultivar	21.5 *	2.61	0.00000 *	0.1	2.61	0.974	12.6 *	2.61	0.00000 *	7.1 *	2.61	0.0001 *	16.5 *	2.61	0.00000 *
Year	72.2 *	2.64	0.00000 *	17.1 *	2.64	0.00000 *	59.7 *	2.64	0.00000 *	12.1 *	2.64	0.00007 *	12.8 *	2.64	0.0004 *
Cultivar * Year	19.5 *	2.61	0.00000 *	0.1	2.61	0.999	9.9 *	2.61	0.00000 *	4.8 *	2.61	0.00009 *	11.8 *	2.61	0.00000 *
Phase GS 71–82 “milk-wax ripeness” 24.05–25.05															
Cultivar	10.1 *	2.61	0.00008 *	0.7	2.61	0.522	12.1 *	2.61	0.00000 *	15.0 *	2.61	0.00000 *	12.1 *	2.61	0.00000 *
Year	53.9 *	2.64	0.00000 *	2.8	2.64	0.058	92.4 *	2.64	0.00000 *	61.3 *	2.64	0.00000 *	6.4 *	2.64	0.0017
Cultivar * Year	8.7 *	2.61	0.00000 *	0.5	2.61	0.801	10.5 *	2.61	0.00000 *	13.0 *	2.61	0.00000 *	6.3 *	2.61	0.0002 *

Notes: F—the actual indicator of the Fisher criterion; F crit.—the critical indicator of the Fisher criterion; *p*—the level of statistical significance (*p* ˂ 0.05). *—mathematically significant influence of the factor on the indicators of the disease development is confirmed

**Table 3:** The results of the correlation analysis of the relationship between the number of spores caught using a spore trapping device and the degree of disease development in wheat crops for three growing seasons from 2019 to 2021.

Cultivar	Powdery Mildew		Yellow Spot		Yellow Rust		Brown Rust	
	R	*p*	R	*p*	R	*p*	R	*p*
Phase GS 40–47 “flag leaf” 1.05–2.05								
Kuren	0.3	0.456	0.1	0.859	0.6	0.099	0	0
Bonus	0.3	0.498	0.1	0.766	0.9 *	0.003 *	0	0
Aksinya	0.1	0.618	0.7 *	0.022 *	0.7	0.057	0	0
Krasnodarskaya 99	0.4	0.264	0.1	0.769	0	0	0.4	0.263
General by groups	0.1	0.674	0.2	0.261	0.6 *	0.001 *	0.3	0.104
Phase GS 61 “early flowering” 12.05–14.05								
Kuren	0.4	0.241	0.9 *	0.0003 *	0.8 *	0.0009 *	0	0
Bonus	0.3	0.440	−0.1	0.952	0.8 *	0.0129 *	0.4	0.249
Aksinya	0.6	0.107	0.1	0.781	0.1	0.978	0.4	0.249
Krasnodarskaya 99	0.7 *	0.021 *	0.9 *	0.0003 *	0.7	0.057	0.8 *	0.005 *
General by groups	0.5 *	0.001 *	0.6 *	0.0001 *	0.6 *	0.0005 *	0.6 *	0.0004 *
Phase GS 71–82 “milk-wax ripeness” 24.05–25.05								
Kuren	0.8 *	0.009 *	0.4	0.258	0.9 *	0.033 *	0.7 *	0.022 *
Bonus	0.8 *	0.006 *	0.1	0.759	0.8 *	0.019 *	0	0
Aksinya	0.9 *	0.003 *	0.5	0.175	0.9 *	0.001 *	0.7 *	0.028 *
Krasnodarskaya 99	0.8 *	0.009 *	0.9 *	0.002 *	0.8 *	0.048 *	0.9 *	0.004 *
General by groups	0.7 *	0.001 *	0.4 *	0.012 *	0.8 *	0.0001 *	0.8 *	0.0001 *
Summarized data for three dates								
Kuren	0.6 *	0.001 *	0.5 *	0.005 *	0.8 *	0.0001 *	0.5 *	0.014 *
Bonus	0.5 *	0.004 *	0.1	0.918	0.8 *	0.0001 *	0.3	0.197
Aksinya	0.3	0.185	0.4	0.058	0.5 *	0.011 *	0.5 *	0.004 *
Krasnodarskaya 99	0.8 *	0.007 *	0.6 *	0.007 *	0.4 *	0.0226 *	0.7 *	0.0001 *
General by groups	0.5 *	0.001 *	0.4 *	0.0001 *	0.6 *	0.0001 *	0.6 *	0.0001 *

Notes: R—correlation coefficient index; *p*—the level of statistical significance (*p* ˂ 0.05); *—statistical significance of data correlation is confirmed.

## Data Availability

Not applicable.
